# Survival and efficacy of entomopathogenic nematodes on exposed surfaces

**DOI:** 10.1038/s41598-022-08605-2

**Published:** 2022-03-17

**Authors:** Jayashree Ramakrishnan, Liora Salame, Ahmed Nasser, Itamar Glazer, Dana Ment

**Affiliations:** 1 Department of Plant Pathology and Weed Research, Agricultural Research Organization (ARO), Volcani Institute, 7505101 Rishon LeZion, Israel; 2 The Robert H. Smith Faculty of Agriculture, Food & Environment the Hebrew University of Jerusalem, 7610001 Rehovot, Israel; 3 Inter-Institutional Analytical Unit, Agricultural Research Organization (ARO), Volcani Institute, 7505101 Rishon LeZion, Israel; 4 Department of Entomology, Nematology and Chemistry Units, Agricultural Research Organization, Volcani Institute, 7505101 Rishon LeZion, Israel

**Keywords:** Surface spectroscopy, Non-model organisms, Environmental impact

## Abstract

Entomopathogenic nematodes (EPN) species differ in their capability to withstand rapid desiccation (RD). Infective juveniles of *Steinernema carpocapsae* are a better adaptable and tolerant than *Steinernema feltiae* or *Heterorhabditis bacteriophora* as, an optimal RH of > 90% is required by *S. feltiae* and *H. bacteriophora* while maintaining RH equivalent to 74% could sustain survival of *S. carpocapsae* under RD. Our findings from infectivity suggest that following application, shrunk IJs are acquired passively by the larvae, probably rehydrate and resume infection within the insect gut. Water loss rate is a key factor affecting survival of *S. carpocapsae* on exposed surfaces. The present study provides the foundation for characterizing mechanism of rapid rate of water loss in EPN. ATR-FTIR is a rapid and reliable method for analysis of water loss. Changes in peak intensity was observed at 3100–3600 cm^−1^ (OH bonds of water), 2854 cm^−1^ (CH stretching of symmetric CH_2_, acyl chains), 2924 cm^−1^ (CH stretching of anti-symmetric CH_2_, lipid packing heterogeneity), 1634 cm^−1^ (amide I bonds) indicate major regions for hydration dependent changes in all EPN species. FTIR data also indicates that, *S. carpocapsae* contains strong water interacting regions in their biochemical profile, which could be an influencing factor in their water holding capacity under RD. ATR-FTIR were correlated to water content determined gravimetrically by using Partial Least square –Regression and FTIR multivariate method, which could be used to screen a formulation’s potential to maintain or delay the rate of water loss in a rapid and efficient manner.

## Introduction

Agriculture sector has seen a paradigm shift in past few years towards green alternatives such as bio-control agents for insect due to strict environmental guidelines^1^. Active infection process by means of tracking and attacking insect pest is an advantageous trait of entomopathogenic nematodes (EPNs) as biocontrol agents. EPN, of the families Steinernematidae and Heterorhabditidae are insect parasites and notable biocontrol agents^2,3^. Free-living, non-feeding third stage infective juveniles (IJs) are predisposed with the ability to track and infect hosts. Infection occurs when IJs gain entry through the natural openings of the host and through the cuticle, release the symbiotic bacteria in hemocoel thereby contributing to killing the host quickly and effectively^4^. The bacteria utilize the host to reproduce, while the multiplying nematodes feed on the bacteria inside the cadaver. The IJs exit the host cadaver, carrying the symbiotic bacteria in their intestine, in search of new hosts^5,6^.

The use of EPN IJs as biocontrol agents are well demonstrated^7–9^. However, several studies have indicated that fluctuating environmental conditions strongly affect EPNs survival and efficacy^10–13^. Thus, EPNs' sensitivity to environmental stresses such as desiccation inhibits its exploitation as effective biological control agents^6,13–15^.

EPNs are mainly applied for controlling soil dwelling pests such as white grubs and fungus gnats^9^. Hence, in soil, which is the natural environment, abiotic stress such as desiccation leads to water removal from their body to occur slowly (within days). Consequently, EPNs are referred as slow dehydration strategist, and their adaptation process to slow desiccation (SD) is also well characterized and recorded^12,16–20^.

Numerous foliar pests such as herbivorous insects and leaf miners are highly susceptible to EPNs under controlled-environment^21^. However, when exposed on surfaces of plant, EPNs encounter rapid desiccation (RD),where water from nematode body is removed within minutes or hours^22–24^. This phenomenon leads to drastic reduction in EPNs activity and viability^14,25,26^. Arthurs et al.^15^ reviewed efficacy of EPN in field applications (including greenhouse) against various pests, identified that performance of EPN varied with the pest target habitat (boreholes, cryptic foliage, exposed foliage) and observed lowest efficacy in exposed foliar applications. Further research identified that, requirement of a thin film of water, high humidity (> 90%) and IJ activity limited by time (approx. 1 h) hampers EPN's natural ability to achieve control of target pest on foliar surfaces^24,27,28^. Hence, the key to successful integration of EPN in foliar surfaces is to be able to achieve a predictable, reliable and consistent control activity against the target pest.

Limited knowledge exists on characterization of RD and its impact on EPN IJ^29–31^. In brief, information on survival trends of EPN in RD, physical mechanisms such as rate of water loss, effect on the physiological, biochemical and molecular protective process are required to understand the adaptation process involved in RD. Hence, it is of paramount importance, to study RD systematically and identify the trends of survival in EPN, characterize the rate of water loss, infectivity patterns at RD to understand the requirements from formulation and identify the optimum microenvironment required for EPN viability and activity in foliar applications.

Understanding the rate of desiccation among IJs of different EPN species, at range of relative humidity (RH) conditions, as well as the influence of these conditions on nematodes efficacy against herbivorous pests will enable to develop formulations to enhance EPN survival and efficacy. We hypothesize that various nematode species would differ in their response to RD. Hence, this may affect the survival, rate of water loss and will result in varying efficacy of the different EPN species toward a defoliator pest. Hence, in the present study the effect of RD on these parameters was evaluated in three commercial EPN species. Since the rate of water loss seem to be a key factor, we evaluated it by the use of two methods for detection; gravimetric measurement and Fourier Transform Infra-Red Spectroscopy (FTIR). We further analysed the correlation between the two methods.

## Results

### Survival and rate of water loss indicates *S. carpocapsae* is better tolerant to RD in comparison to *S. feltiae* and *H. bacteriophora*

**Figure 1:**
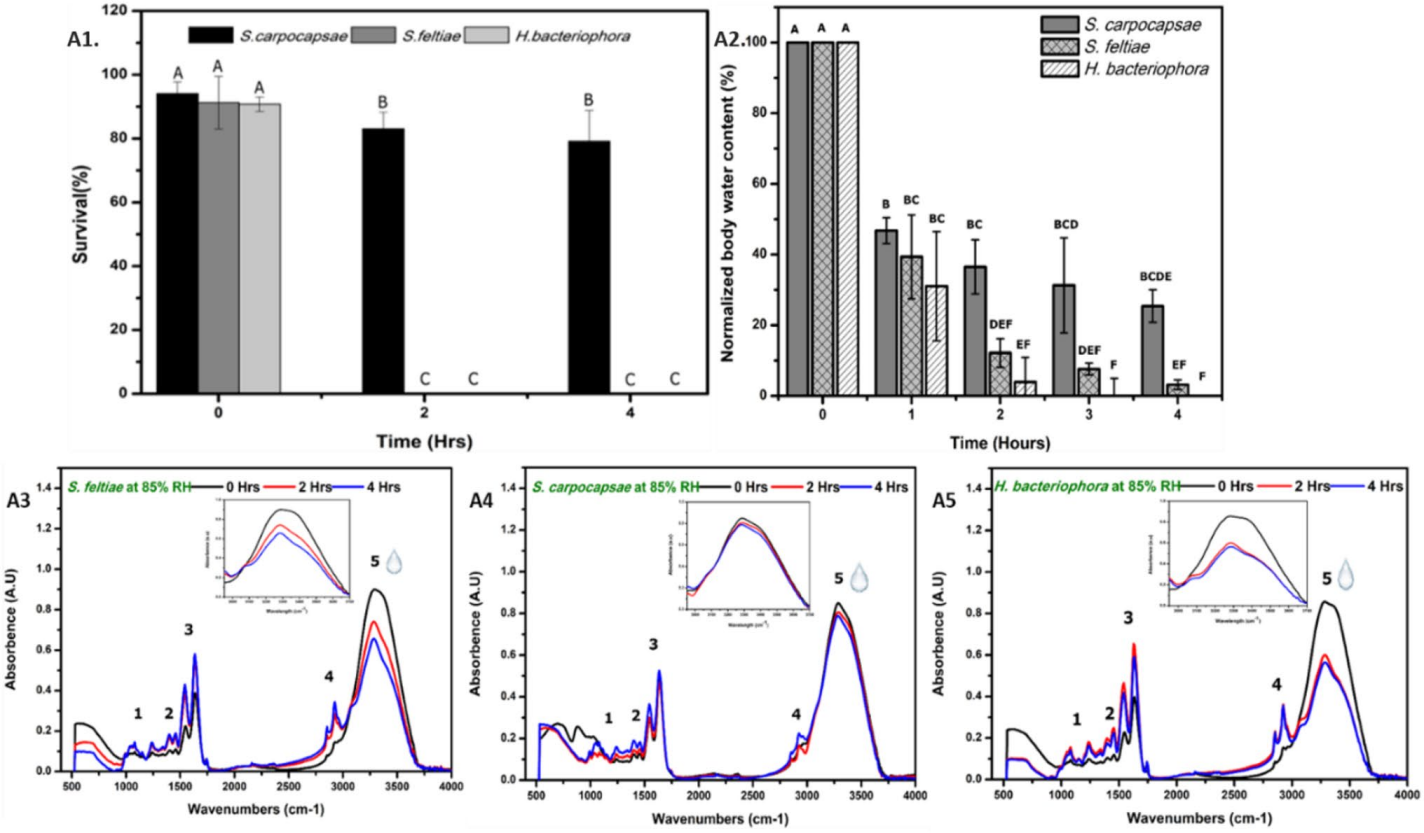
EPNs species (*S. carpocapsae* (SC), *S. feltiae* (SF), *H. bacteriophora* (HB)) rapidly desiccated at 85%RH. Survival (%) (**A1**) and gravimetric rate of water loss (**A2**) at 85%RH. (**A3**–**A5**) Comparison of FTIR spectra depicting the water peak of rapidly desiccated *S. feltiae, S. carpocapsa*e, *H. bacteriophora* at 85%RH for 0, 2 and 4 h. Inlet in each figure represents the peak of OH (water)/NH bonds. The numbers inside the spectra represents the bands that were strongly affected due to different humidities (see Table S1 for assignment of functional groups).

EPN species differ in their response to RD at 85% RH (Fig. 1-A1). More than 80% of *S. carpocapsae* survived in comparison to complete mortality observed in *S. feltiae* and *H. bacteriophora* at 2 h into RD*.* Hence, rapidly desiccated *S. carpocapsae* survival was significantly different from *S. feltiae* and *H. bacteriophora* (*p* < 0.0001) at 2 and 4 h. However, *S. feltiae* and *H. bacteriophora* were not significantly different from each other at 0, 2 and 4 h (*p* = 1.0*, p* = 0.99 and *p* = 1.0) respectively.

Water content of *S. carpocapsae, S. feltiae and H. bacteriophora* varied with time and among species. The water content of EPN species determined by gravimetric measurements was significantly different and varied with time (Fig. 1-A2 and p < 0.0001). All EPN species lost ca. 50% of their water content within the first hour of exposure to RD. After 1 h, *S. carpocapsae* had the lowest reduction in water content (54%), *H. bacteriophora* had the highest reduction (69%). Additional exposure for 1 h at 85%RH resulted in further reduction of 30–33% and 25–27% water content in *H. bacteriophora* and *S. feltiae* respectively, whereas *S. carpocapsae* lost only 10% of water content. In addition, at 4 h, *S. carpocapsae* had a fourfold higher water retention (approx. 30% water content) and significantly different in comparison to *S. feltiae* (ca.7% water content) and *H. bacteriophora* (p < 0.001). Among the tested EPN species, *S. carpocapsae* was able to adapt and maintain water content reduction from 1 to 4 h of RD at 85% RH as also indicated by its survival.

**Table 1:** Saturated salt solutions used to achieve relative humidity.

Salt	Relative humidity attained (%)
Potassium chloride (KCl)	85
Sodium nitrate (NaNO_3_)	74
Sodium nitrite (NaNO_2_)	64
Calcium nitrate (CaNO_3_)	55
Potassium carbonate (K_2_CO_3_)	43

**Figure 2:**
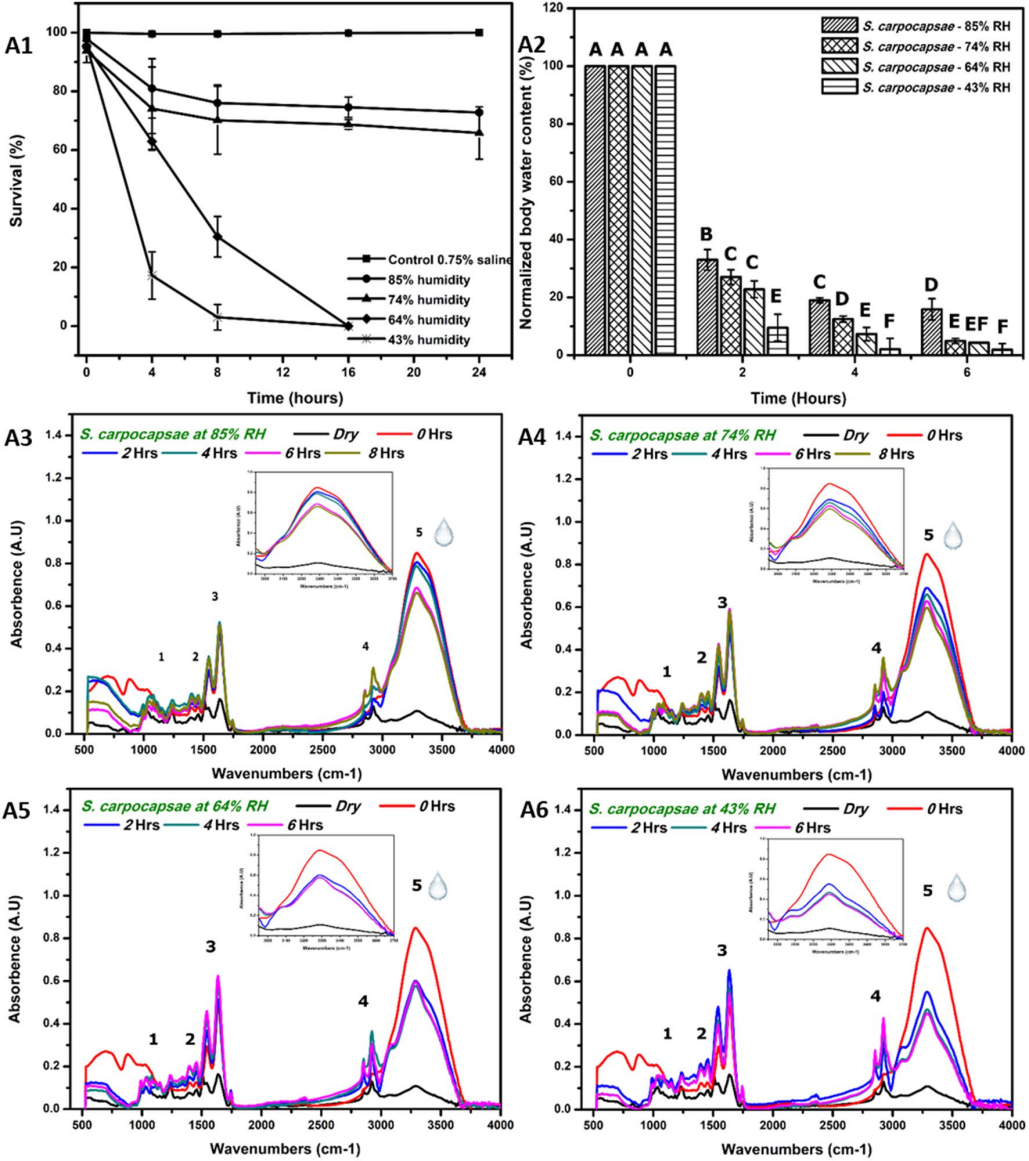
Rapid desiccation of *S. carpocapsae* at 85, 74, 64 and 43% RH. (**A1**) In-vitro survival (%) of *S. carpocapsae* at different humidity for 0, 4, 8, 16 and 24 h. (**A2**) Gravimetric rate of water loss of *S. carpocapsae* rapidly desiccated for 0, 2, 4, 6 h at different RH. (**A3**–**A6**) FTIR fingerprint spectra of rapidly desiccated *S. carpocapsae* at 85, 74, 64 and 43%RH for 0, 2, 4 and 6 h. Inlet in each figure represents the peak of OH (water)/NH bonds. The numbers inside the spectra represents the groups strongly affected due to different humidities (see Table S1 for assignment of functional groups). Survival (%) (n = 360) and gravimetric water loss (%) (n = 156) were arcsine square-root transformed and subjected to comparison of standard least squares by REML. When ANOVA *F* was significant (p < 0.05), means were compared. Mean was compared using Tukey test. Different alphabets represent significant difference.

### Survival of *S. carpocapsae* under low humidity

In vitro survival of rapidly desiccated *S. carpocapsae* at lower humidities of 85, 74, 64 and 43% RH was estimated. The experiment was repeated four times with five technical replicates and the humidity was maintained as mentioned in Table 1. Response of *S. carpocapsae* survival varied with different humidities and was significantly different from control (*p* < 0.05) (Fig. 2-A1). Survival of *S. carpocapsae* IJs varied with time and was found to be significantly different (*p* < 0.001). At 85% and 74% RH, *S. carpocapsae* exhibited the highest survival (> 80% and > 75% respectively) while at 43% humidity the sharpest decline in survival to 20% within 4 h was observed. This indicates lower tolerance capability of *S. carpocapsae* towards harsh humidities below 60% RH. Two-fold reduction (20%) in survival of *S. carpocapsae* was observed at 43% RH in comparison to 55%RH with an observed 58% survival (Fig. S1). The least tolerable humidity range for *S. carpocapsae* at 23 °C was found to be between range of 55–43% RH with a significant difference within this humidity range (p < 0.0001).

Gravimetric water content of *S. carpocapsae* at different RH were significantly different with time (p < 0.001) (Fig. 2-A2).Water content in *S. carpocapsae* reduced to approximately 40, 25, 18 and 1% within 2 h of exposure at 85, 74, 64 and 43 RH respectively. At 85%RH, water content in *S. carpocapsae* reduced gradually from 40 to 12% and at 74% RH, reduced from 25 to 8% over the time of 2–6 h. In comparison, water content at 64 and 43% RH reduced drastically to 20% and 1% within 2 h of exposure.

### Efficacy of *S. carpocapsae* at different humidities

**Figure 3:**
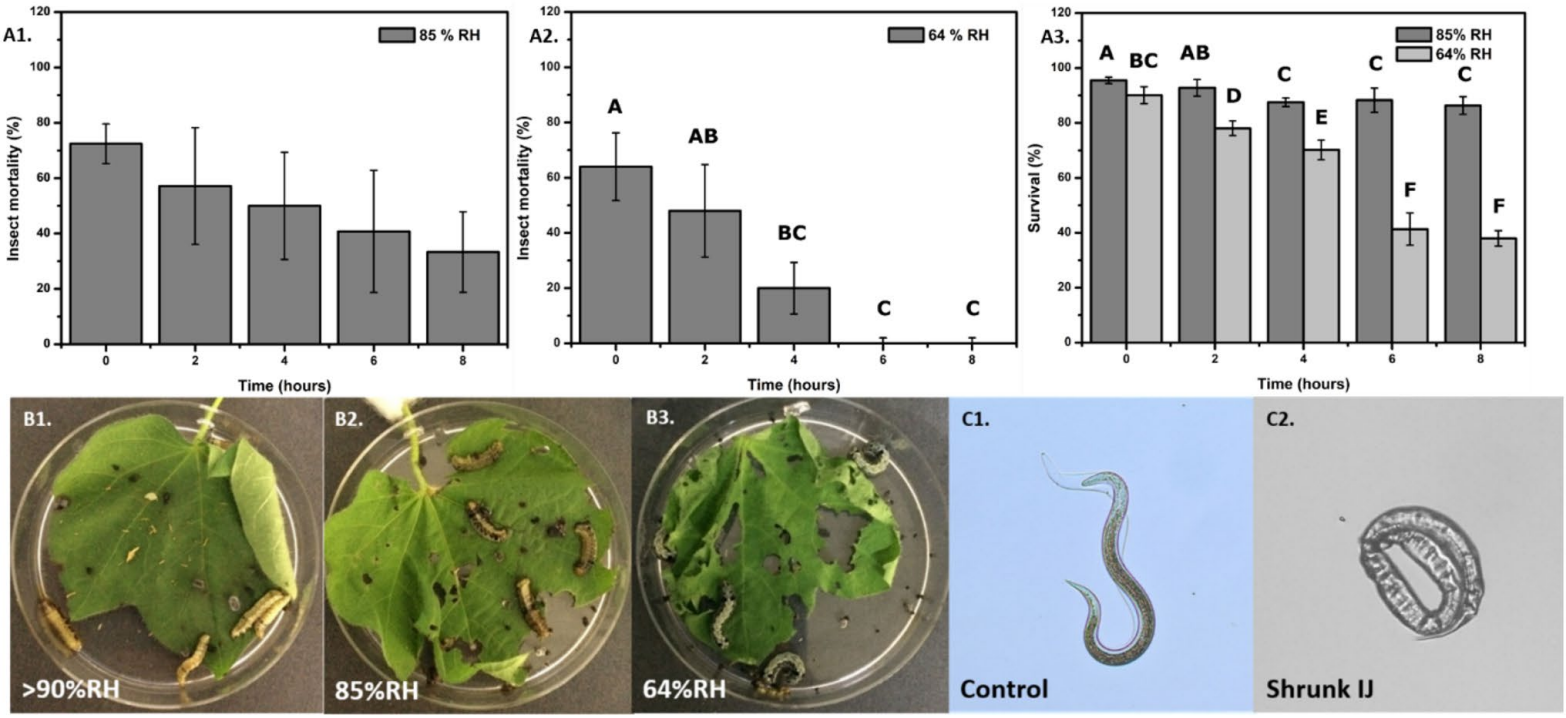
Insect mortality in rapidly desiccated *S. carpocapsae* at 85 and 64% RH. Insect mortality of late 4th instar larvae of *Spodoptera littoralis* against *S. carpocapsae* applied at a concentration of 5000 ± 150 IJs/leaf and incubated for 48 h at 85%RH (**A1**) and 64%RH (**A2**). (**A3**) Nematode survival on leaf at 85 and 64% RH over 0, 2, 4, 6 and 8 h. (**B1**–**3**) Images of experimental set up. (**C1**,**2**) Images exhibiting fresh/control and shrunk *S. carpocapsae* IJ on leaf after 1.5 h of RD under 44% RH.

Nematode exposed to RD, followed by introduction of host and its influence on efficacy of IJs was assessed. After application of IJs on leaf and exposure to 85%, 64% RH, larvae starved for 1 h were added at 0, 2, 4, 6, and 8 h (Fig. 3-B1–B3) and sealed with mesh screen plates.

Relative humidity had an impact on the efficacy of *S. carpocapsae*. The efficacy against late fourth larval instars of *S. littoralis* decreased with decrease in RH and observed decreased efficacy with increase with time. Although no statistical significance in mortality at 85%RH was observed (Fig. 3-A1), it is critical to note the nematode survival at the time of larval addition and correlate it to the mortality observed (Fig. 3-A1 and A3). A significant (p < 0.0001) impact in efficacy was observed at the lower humidity (Fig. 3-A2). At optimal humidity, complete mortality was observed in comparison to 72 and 64% insect mortality observed at 85 and 64%RH for 0 h. However, with time, mortality reduced drastically from (ca.) 64 to 20% between 2 and 4 h at 64%RH, while decline in survival from remained consistent (Fig. 3-A2 and A3). In addition, no mortality was observed at 6 and 8 h of desiccation, while survival remained at ca. 40%.

### ATR-FTIR as a rapid, efficient alternative tool for water content determination

EPN species were prepared for ATR-FTIR according to San -Blas et al.^37^. FTIR fingerprint spectra of EPN species varied in response to RD at 85% RH (Fig. 1-A3–A5).

The peak at 3100–3600 cm^−1^ generally represents water and NH bonds in nematodes. The reduction of peak height and intensity at this region in EPN species represents the effect of hydration of nematodes. In accordance with the gravimetric water loss, *S. carpocapsae* accounted for the slowest reduction while *H. bacteriophora* had the highest reduction in water content within 4 h. In addition, in FTIR, several regions such as 2854 cm^−1^ (acyl chains), 2924 cm^−1^ (lipid packing), and 1634 cm^−1^ (amide bonds) indicate major regions for hydration dependent changes in RD was evident from two-way ANOVA test (Table S1). Interestingly, spectral regions assigned to lipid packing changes (Region 4 in Fig. 1-A3–A5) at 2854, 2924 cm^−1^ indicate this region were least affected in *S. carpocapsae* in comparison to *S. feltiae* and *H. bacteriophora* within 4 h into RD (Fig. S2). Principal component analysis (PCA) (Fig. S3) indicates their differential distribution of EPN species with time at 85% RH under RD. *S. feltiae* and *H. bacteriophora* were grouped similarly while *S. carpocapsae* was grouped separately indicating a difference in the response to RD.

### Water content of *S. carpocapsae* at 85, 74, 64 and 43% RH by ATR-FTIR

Nematodes (*S. carpocapsae*) were prepared as described and desiccated at 85, 74, 64 and 43%. Spectra at varying time points and humidity revealed a continuous loss of water content from nematode during RD (Fig. 2-A3–A6) thus revealing reductions in peak intensity at 3300 cm^−1^ (OH bonds) from 0 to 2 h at low humidity (64 and 43% RH) (Fig. 2-A5 and A6) and 2 to 6 h at high humidity (85 and 74% RH) (Fig. 2-A3 and A4). This phenomenon was in accordance with gravimetric water loss. PCA (Fig. S4) of *S. carpocapsae* at different humidity indicate the response to RD at higher humidity is similar and lower humidity could be grouped together. Thus, ATR-FTIR is rapid, effective and non-invasive technique to identify hydration dependent changes in EPN.

**Figure 4:**
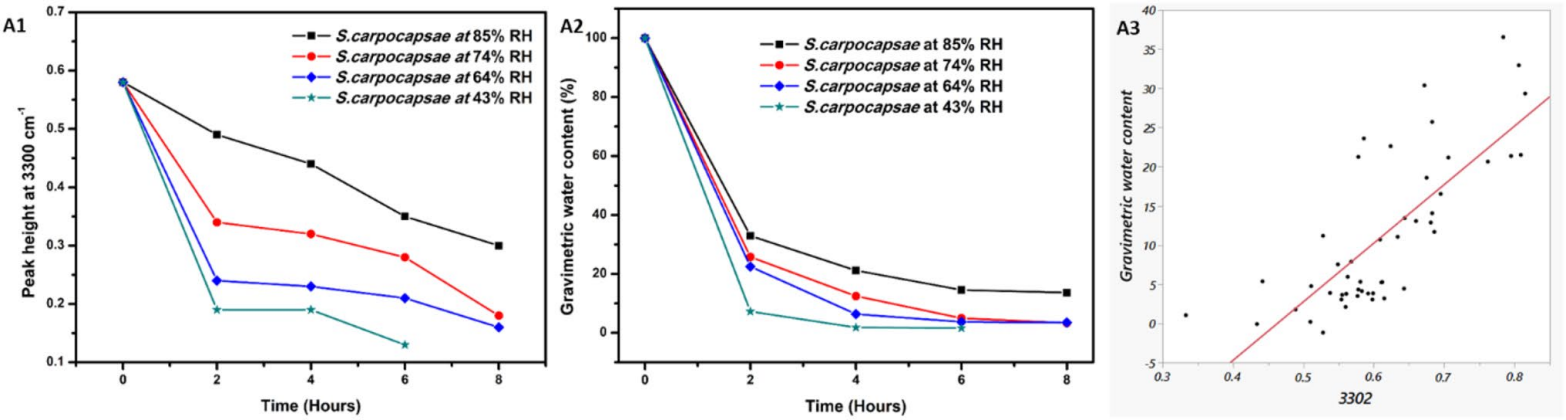
FTIR univariate analysis of rapidly desiccated *S. carpocapsae* at 85, 74, 64 and 43%RH. (**A1**) Peak height obtained from wavelength of 3301 cm^−1^ and (**A2**) measured gravimetric water content at different humidity. (**A3**) Bivariate fit of data obtained from Gravimetric water content and 3302 cm^−1^ (R^2^ = 0.58).

### FTIR univariate analysis

FTIR spectra at peak 3300 cm^−1^ (indicative for NH bonds and region of 3100–3600—indicative for water) record a continuous decrease in peak height (Fig. 4-A1) while the dry spectra of RD nematodes at 3100–3600 cm^−1^ indicate that peak differences observed at differing humidities were an effect of water (Fig. 2-A3, indicated by black line). Hence, FTIR data was interpreted by Univariate analysis.

This univariate approach indicated that, at higher humidity (85 and 74%RH) the relative water content was two-one fold higher in comparison to gravimetric measurements respectively (Fig. 4-A1 and A2). To confirm, we identified if the fit of the data for high humidity was acceptable (Fig. 4-A3). The fit of the data (R^2^) ranged between 0.78–0.55 from 3560–3100 cm^−1^ while fit of data at 3301 cm^−1^ was 0.58. In addition, fit of data (R^2^) obtained from area under curve (AUC) compared to gravimetric measurements was 0.60. These results indicate that the effect of water loss cannot be completely explained by the region of 3600–3100 alone and changes in other spectral regions in IR (i.e.) 4000–400 cm^−1^ (Fig. 2-A3–A5) should be considered for predicting water content as well.

**Figure 5:**
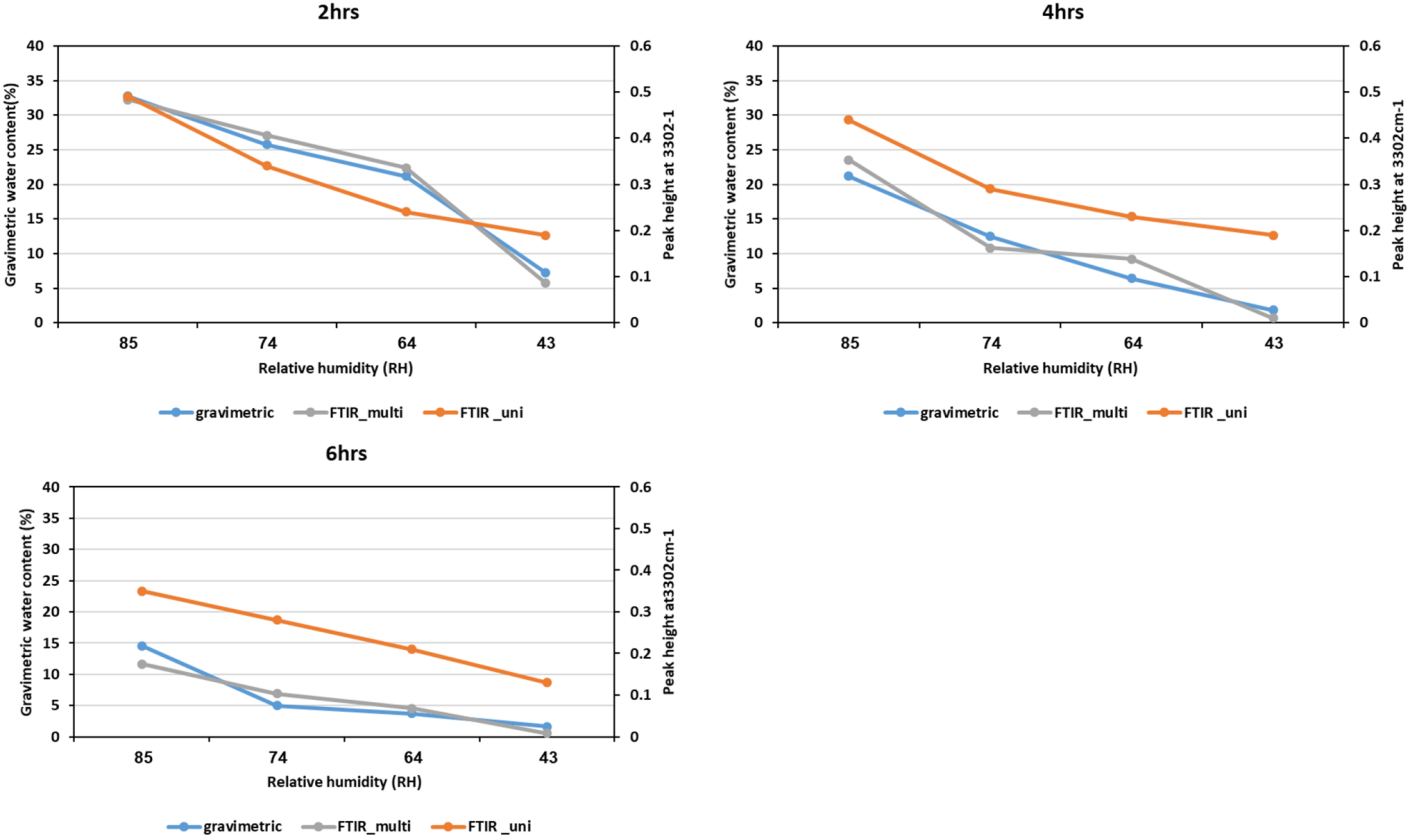
Comparison of water content in rapidly desiccated *S. carpocapsae* at 85, 74, 64 and 43%RH arising from Gravimetric measurements, FTIR univariate and predicted by FTIR multivariate method using PLS-R. The correlation coefficient (R^2^) of FTIR multivariate to gravimetric measurements was 0.93.

### FTIR multivariate analysis

Exploiting the data obtained from whole FTIR spectra (4000–400 cm^−1^, Fig. 2-A3–A6) and model water content with corresponding gravimetric measurements as reference to calculate multiple partial least squares-regression models to perform their validation.

The spectral regions and quality indicator (R^2^-coefficient of determination) for cross validation  was used to calculate the PLS-R model. The average number of PLs components were inferior to 7 and the coefficient of determination was greater than 0.93, confirming the quality of the selected models. Hence, the data arising from the predictions were plotted against the gravimetric water content and FTIR univariate (Fig. 5) and the comparison of actual vs predicted values obtained from Gravimetric estimations and PLS-R predicted values respectively are indicated in (Fig. S5). It is important to notice that, the predictions arising from FTIR PLS-R model fits very well with the experimental gravimetric measurements (R^2^ > 0.93) highlighting the requirement of FTIR multivariate.

## Discussion

We evaluated and correlated water loss through RD and survival of three EPN species. Two methods were applied: gravimetric, and spectral-based ATR-FTIR. Gravimetry is the standard reference method for water-content analyses, whereas ATR-FTIR enables rapid measurements of biological specimens^32^, and is the best approach to studying hydrated and dried samples, such as cells^33^. FTIR has been extensively used to study hydration-dependent changes in desiccated biological materials, to identify, e.g., the survivability of pollen^34^, the role of trehalose in preconditioned *Caenorhabditis elegans*^35^ and the ability of lipid bilayers to increase their affinity for trehalose during slow desiccation^36^. However, in EPNs, FTIR application has been limited to EPN species identification and analyses of biochemical composition^37,38^. In this study, identifying the rate of water loss in nematodes was a basic prerequisite to evaluate the effect of RD on nematode physiology. Hence, ATR-FTIR was used to identify the water loss from nematodes. Moreover, we wanted to correlate the gravimetric and FTIR measurements based on the biochemical profile of EPN species during RD at different RH and times, and on differences in the 3100–3600 cm^−1^ peak assigned to hydroxyl (–OH) vibrations of water^39,40^.

The tolerance of different EPN species to RD conditions was compared, and *Heterorhabditis* and *S. feltiae* were found to be poor survivors. These findings are similar to those of Liu et al.^11^, where their survival was estimated to be 15 min at RH ≤ 88%. Other studies have shown that the EPN species studied in this work differ in their survival rates under RD^14,29,30,41^. The better survivability of *S. carpocapsae* found here under RD at moderate RH of 74–85% has been noted previously as well^14,26,29,42,43^. Under the environmental conditions of RD used in this study, > 60% water loss within the first 2 h was detrimental to the survival of both *S. feltiae* and *H. bacteriophora*, in accordance with Patel et al.^41^. In contrast, *S. carpocapsae* was able to survive a water loss of > 50% in the first 2 h. FTIR spectra also indicated a decrease in absorbance of the water peak at 3300 cm^−1^ at different time points in accordance with RH. PCA of the FTIR data for *S. carpocapsae*, *S. feltiae* and *H. bacteriophora* also indicated their differential distribution with time under RD at 85% RH. *S. feltiae* and *H. bacteriophora* clustered together, while *S. carpocapsae* was grouped separately, indicating a different response to RD.

The advantageous survival capacity of *S. carpocapsae* has been attributed to factors such as water permeability of the cuticle and other physiological adaptations, among them epicuticle thickness and chemical composition^42,44–46^. Interestingly, *S. carpocapsae* had thinner cuticle and epicuticular layers (273 nm and 18 nm, respectively) than *S. feltiae* (374 nm and 33 nm, respectively)^41^; data for *Heterorhabditis* were not found in the literature. Somvanshi et al.^47^ and Yaari et al.^17^ showed that EPN species with higher desiccation tolerance, such as *S. carpocapsae*, are characterized by the constitutive expression of genes known to be involved in desiccation-tolerance mechanisms. Recent findings from Gade et al., also indicate that, related organisms such as *C. elegans* possess a common survival framework in response to varied abiotic stress^48^. These results relate to preconditioning while our results are based on rapid desiccation. Thus, the results in the present study imply an intrinsic mechanism controlling the rate of water loss in *S. carpocapsae*. Hence, the higher tolerance of *S. carpocapsae* IJs to RD could also be attributed to bioprotectant processes. However, this hypothesis requires further investigation  on the molecular and biochemical changes occurring during RD.

Our results showed that the ability to manage water-loss rates depends strongly on the surrounding RH conditions. The IJs of *S. carpocapsae* were able to gradually reduce their rate of water loss at higher humidities (85% and 74% RH) from 0 to 6 h. However, at lower humidities (64% and 43% RH), poor survival was observed within 4 h, indicating that these IJs could not retain their internal water under these low RH conditions. This implies the requirement of a minimum range of time for controlling the rate of water loss and maximizing the intrinsic control and protective mechanisms. Findings of survival and measurements of the rate of water loss by both gravimetric and FTIR measurements in this study indicated that the first 4 h of RD exposure are critical for protective mechanisms. Differences in water content of *S*. *carpocapsae, S. feltiae* was observed by Patel et al.^41^ from the current study, within 1 h and 30 min of RD at 85% RH respectively. The variation in results could be due to: (a) different methods of water-content estimation; (b) the substrate’s ability to hold moisture. Thus, substrate also plays a vital role in influencing the survival of EPNs under RD.

Our FTIR results indicated that *S. carpocapsae* can maintain water at 85% RH, in comparison to *S. feltiae* and *H. bacteriophora* thus reiterating the water-holding capacity of *S. carpocapsae* at high humidity (85–74% RH). An interesting observation from FTIR of EPN species was in the region of 2854, 2924 cm^−1^. As suggested by Erkut et al., (2011) this region is attributed to lipid chain packing heterogeneity (2924 cm^−1^) and reduced lipid-packing density (2854 cm^−1^). These regions in *S. carpocapsae* were unaffected by water loss at 85% RH, even though gravimetric water content was ca 38%. This suggests strong water-interacting regions in the biochemical profile of *S. carpocapsae*, such as the chemical composition of lipid-rich epicuticle layer and the glycoprotein-rich surface coat, which could all be influential factors in the high tolerance to RD^49–51^. Hence, if these regions are unaffected, it is indicative of the effect of water permeability. Selvan et al.^52^ identified differences of chemical composition (i.e.) higher saturated lipids in *S*. *carpocapsae* in comparison to higher unsaturated lipids observed in *S. feltiae*. This could contribute to water retention in *S. carpocapsae* as membranes made of saturated fatty acids are less permeable to water and salts. However, this hypothesis requires further research. Thus, FTIR in particular enables to compare chemical components/functional groups of EPN under the same state of desiccation. Celino et al.^53^ identified and correlated gravimetric and FTIR data of plant fibers using univariate and multivariate methods. In the univariate method, relative water content was estimated solely as a representative of variations arising from the absorbance of hydroxyl groups at 3100–3600 cm^−1^^46^, i.e., spectral data obtained from one peak that is characteristic of vibration of hydroxyl bonds in water (at 3300 cm^−1^). Hence, the large difference in peak height observed for dry and RD *S. carpocapsae* could indicate interactive regions of hydrogen bonds. Hence, we measured the peak height of the samples after subtraction from the dry spectra. This enabled identifying changes induced only by loss of hydroxyl groups. The comparison of peak height and gravimetric measurements yielded differences that are best explained by Park’s model, attributing them to the ability of water molecules to form clusters at higher humidity^54^. Thus, the whole IR spectrum of RD nematodes was also impacted by changes in humidity and time, from spectral regions 1600–500 cm^−1^, indicating that a multivariate approach is suitable for accurate monitoring of water content, as also noted by Celino et al.^53^. Our analysis inferred that the multivariate method is better correlated to gravimetric measurements than the univariate method. This could be attributed to the consideration of all regions from 4000 to 400 cm^−1^ to obtain better predictive models to quantify water content. In addition, the R^2^ value of 0.93 in the cross-validation of the obtained data by PLS-Regression method indicated a very high correlation between predicted and gravimetric measurements.

Another interesting observation is that insect mortality due to nematode infection accrued, even after the first 4 h at 85 and 64% RH on the leaf surface, whereas IJs tended to cease movement and shrink within 30–40 min of exposure to low RH. Technically, even one IJ could cause mortality however; we observed a drastic reduction in mortality at 64% that might indicate that low humidity condition has an effect on the nematode efficacy irrespective of the concentration. We therefore presume that the positive infectivity is due to rehydration of IJs in the insect gut and resumption of infectivity. These findings are in agreement with Glazer^14^, who identified nematode shrinkage after 45 min on the leaf surface while retaining efficacy, indicating route of entry through consumption of treated leaves by the insect. This ability of desiccated IJs to rehydrate inside the insect gut and resume infectivity was also reported by Wojicik and Georgis^55^. Recent research on *Diptera* has also indicated the ability of IJs to infect larvae by passage to the haemolymph from the gut through the peritrophic membrane^56^. This would indicate that motility of rehydrated IJs inside the insect gut is critical for successful infection. Hence, at 64% RH, no insect mortality was recorded despite the 40% survival rate of IJs; we assume that these IJs were not able to enter the haemolymph or were rendered inactive during rehydration.

The results of the current study lead to the interesting assumption that host behaviour on the foliage is crucial to appropriately pair the right method of EPN application, given that infectivity is still positive under non-active penetration (by ingestion) of EPNs. In turn, this could mean that requirements for formulations have to be pre-established, depending on the host. For example, in the case of active, polyphagous feeders such as *Spodoptera*, a formulation requirement would be to maintain a steady rate of water loss with rapid rehydration. This is in contrast to other types of foliage pests, such as leaf miners or sucking pests, where it is necessary to maintain the activity of IJs to ensure host tracking and successful infection.

## Conclusion

Overcoming the low viability and erratic infectivity of EPNs on foliage through formulations has been a focal point of research since 2000^24,57–63^. Here, we focused on the physical parameters of RD as well as its effect on IJ efficacy on exposed surfaces. ATR-FTIR is a rapid and reliable method for analysis of water loss. Changes in peak intensity were observed at 3100–3600 cm^−1^ (OH), 2854 cm^−1^ (symmetric CH stretching CH_2_, acyl chains), 2924 cm^−1^ (antisymmetric CH stretching CH_2_, lipid packing) and 1634 cm^−1^ (amide I bonds), indicating major regions for reflecting hydration-dependent changes in all EPN species. ATR-FTIR measurements were correlated to water content determined gravimetrically using partial least square regression and FTIR multivariate method, and this could be used to screen a formulation’s potential to maintain or delay the rate of water loss in a rapid and efficient manner. The present study provides the foundation for characterizing rapid rates of water loss. Our observations indicate that following exposure to RD, IJs cease moving and shrink within 20–40 min; however, insect mortality due to nematode infection continues to occur, even after 2 h of exposure. Our findings suggest that shrunken IJs are acquired passively and probably rehydrate and resume infection within the insect gut. Hence, it can be extrapolated that although active infection processes (of IJ) are a sought-after trait in EPNs, on foliage, host behaviour also influences the dynamics of the infection process. From this perspective, the nature of EPN behaviour during RD is crucial for infectivity. Our findings provide the basis for the development of new formulations that will enhance nematode survival and activity during the critical period required to ensure efficacy. Further fundamental biochemical and molecular research is required to maximize formulation functionality for survival and infectivity of EPNs on foliage.

## Materials and methods

### Nematode source

EPN response to RD was characterized by studying the commercially available lines of *Steinernema carpocapsae*, *Steinernema feltiae* and *Heterorhabditis bacteriophora* obtained from e-nema (e-nema, GMBH, Schwentinental, Germany). IJs were reared in last instar larvae of *Galleria mellonella* according to Stock and Kaya^64^. The cadavers were transferred to modified White trap^65^ and incubated at 23 °C. The emerging Infective Juveniles (IJ) were collected and stored at 8 °C for *Steinernema* and 14 °C for *Heterorhabditis* until used further for bioassays. Every 2 weeks fresh stock of IJs were propagated.

### In-vitro EPN survival at high relative humidity

Survival of EPN species (*S. carpocapsae*, *S. feltiae* and *H. bacteriophora),* rapidly desiccated at 85% humidity was estimated and compared. Approximately 5000 IJs in 1 ml of each of the 3 nematodes lines were vacuum filtered on filter paper (Whatman no.1, 55 mm Diameter), dried under laminar airflow (LAF) chamber for 10 min, to remove excess water. Samples were transferred to desiccator at 85% RH (maintained with saturated salt solutions as outlined in Table 1^66^), 23 °C and recorded continuously for humidity by data logger (LOG32TH and SSN23E). Nematodes suspended in saline and incubated at 23 °C served as control. Samples were collected at 0 (immediately after filtration), 2 and 4 h of RD. Samples from filter paper (approximately 0.6 cm × 2 cm) were cut, suspended in saline solution and incubated overnight for assessment of nematodes survival rate. This was done by random counting the number of live or dead among approximately 200 IJs under binocular (Olympus, SZH10, 30× magnification). IJ were scored alive on active movement and on response to probing. Data was used to calculate survival rate as percentage of total number of nematodes. The experiment was repeated twice with 5 replicates per treatment.

### Survival of *S. carpocapsae* under low humidity

In-vitro survival of rapidly desiccated *S. carpocapsae* at lower humidities of 85%, 74%, 64% and 43% RH was estimated. The nematodes samples were prepared as outlined in previous section. Samples from filter paper (0.6 cm × 2 cm) were cut, suspended in saline solution and incubated overnight for survival rate. Survival rate was determined as outlined previously. The experiment was repeated four times with five replicates per treatment.

### Evaluating EPNs water content by gravimetric method

Water content of EPN species during RD was estimated using Gravimetric method as described by Erkut et al.^35^ with slight modifications. IJs at an amount of 50,000 IJ suspended in 120–150 µl saline were vacuum filtered on 1.3 cm diam. filter paper and dried under laminar airflow for 20 min. The obtained samples following this procedure were regarded as 0 h. samples were weighed (0 h) and transferred to desiccator maintained at specified humidities (85, 74, 64, 43% RH). Control treatments consisted of 120–150 µl of saline solution that were processed as mentioned above to remove the effect of residual water in filter paper. vacuum filtered and dried. All samples and controls were weighed at various time points (1, 2, 3 and 4 h at 85% and for *S. carpocapsae* at lower humidities*—*2, 4, 6, 24 h at 85, 74, 64, 43% RH). Dry weight was estimated by oven drying at 90 °C for 1 h. Weight of water were calculated by subtracting the dry weight of nematode samples from the estimated weight at different time points. Further, the weight of the control samples were also negated to remove the effect of residual saline on the water loss. Hence, the water content of nematodes estimated in weight (mg) and was expressed in percentage (%) as Normalized body water content (NBWC) using the following formulae:

$$NBWC \left(\%\right)=\frac{{W}_{t}}{{W}_{0}}\times 100$$

where, "W_t_" represents weight of samples at different time point (e.g.: 0, 2, or 4 h). "W_0_" is the initial weight at 0 h.

The experiment was repeated 3 times with three technical replicates.

### Attenuated total reflectance-Fourier transform infrared spectroscopy (ATR-FTIR)

#### Water content determination of EPNs by ATR-FTIR

FTIR spectra of EPN species during RD were identified using ATR-FTIR as described by San-Blas et al.^37^ with slight modifications. Nematodes at a concentration of 50,000 IJ/150 µl were vacuum filtered, dried under LAF for 15–20 min and transferred using spatula to a glass slide gently. Samples were assessed for water content at 0, 2 and 4 h at 85% RH (EPN species) and 0, 2, 4, 6, 8 h at 85, 75, 64, 43% RH (*S. carpocapsae* at different humidity). Glass slides with nematodes were inverted, placed on ATR-FTIR (Bruker, Germany), pressure clamp was applied. The spectra were collected from 4000 to 400 cm^−1^, 4.0 cm^−1^ spectral resolution and 32 scan. Spectra was also collected for oven dried samples at 90 °C for 1 h. The experiment was repeated twice with three technical replicates per sample.

#### FTIR spectra pre-processing

All the spectra were recorded to the background spectra. For the multivariate analysis, the raw spectra were automatically corrected for ambient CO_2_, smoothed by adjusting the data spacing at 16 cm^−1^. All these treatments were achieved using integrated functions of the instrument (Nicolet IS5, Bruker Optics, Germany). Pre-processed spectra were exported as text files for file format modification on Microsoft Excel. The spectral data were further analysed by performing multivariate analysis Principal Component Analysis (PCA) using the JMP15^53^.

#### FTIR univariate analysis

The spectra of RD nematodes at the region of 3600–3100 cm^−1^ were subtracted from the dry nematodes. The resultant peak at 3300 cm^−1^ was measured for peak height using the peak height tool. This region is considered to be representative of OH bonds. All these changes were carried out in the Omnic software. The measured peak heights were plotted against the gravimetric water content. The peak area of region 3100–3600 cm^−1^ was measured by origin software (OriginLab Corporation, Northampton, MA, USA) version 8 by fit single peak with built in guass function after baseline correction. The baseline was corrected by fitting method under exponential curve and by finding anchor points for detecting the baseline.

#### FTIR multivariate analysis

Calibration and cross validation was conducted using Partial least squares-regression (PLS-R). Firstly, the pre-processed infrared spectra were Partial least squared against the reference values obtained from Gravimetric water content estimation. At this step, a formula is generated for each wavelength to equate to the gravimetric measurements obtained. This step also allowed building models for prediction of formulae based on the number of correlated factors by NIPALS and with number of factors ranging from 5 to 7. The number of factors identified were based on percentage of variations that was able to explain the differences between gravimetric measurements (Y response) and FTIR wavelengths (X response). The number of factors identified was 5 based on the percent variations. This factor was able to explain 94% variations in FTIR based on 95% variation in gravimetric measurements. Secondly, a cross validation was performed by regression method using the lasso estimation and holdback of 0.33% of the samples (i.e.) 11 spectra were randomly selected by the software to perform the cross validation. This procedure allowed finding models with high correlation (R^2^).

### Efficacy of *S. carpocapsae* at different humidities

Nematodes were assayed for their activity against the 4th instar of the foliage feeder *Spodoptera littoralis* (Egyptian cotton leafworm) larvae. Insect colony was continuously maintained as described in Birnbaum et al.^67^. The larvae were used 16–19 days post hatching to ensure homogenous development. The experiment was carried out on 55 mm petri plates with holes to insert the petiole of the cotton leaf. To keep the leaves fresh, the tip of the petiole was wrapped with moist cotton (Fig. 3-B1–B3). Each leaf was applied with 5000 nematodes spread with loop, allowed to dry under hood for 15–20 min. Control plates were sealed with regular petri lids, wrapped with Parafilm and sealed. Mortality was assessed after 48 h. and gently transferred to desiccators maintained at 75% RH and 55% RH at 24 ± 1 °C. A shift in + 10%RH was recorded due to the presence of leaf. The nematodes were exposed to each RH for 0, 2, 4, 6 and 8 h before exposing it to the insect larvae. In addition, nematodes were also observed for their behaviour of shrinking at the time of larval addition (Fig. 3-C1 and C2).

Five instar larvae were added, closed with mesh screened plates and sealed with Parafilm. The plates were maintained in the desiccators and incubated at 24 ± 1 °C. Insect mortality was determined after 48 h. Each treatment contained five technical replicates (dishes). An extra replicate was monitored for evaluating the survival of the nematodes. These samples were evaluated for nematodes' survival following overnight incubation. Experimental protocols involving plant materials was conducted in accordance with institutional, national, and international guidelines and legislation. The Akala variety cotton plants were grown under greenhouse conditions for 3 months and used for experiments and permissions were obtained from the Agricultural Research Organization.

### Statistical analysis

All statistical analyses were undertaken using JMP version 15 pro (SAS Institute Inc.). Comparisons of survival and gravimetric water content bioassays were carried out on the arcsine‐transformed proportions of live nematodes and normalized body water content (%). They were subjected to an analysis of standard least squares by restricted maximum likelihood (REML) followed by Tukey's HSD test for multiple comparisons among means. Survival (%) (n = 90) and gravimetric water loss (%) (n = 72) were arcsine square-root transformed and subjected to two way ANOVA and comparison using standard least squares by REML method respectively. When ANOVA *F* was significant (p < 0.05), means were compared. Mean was compared using Tukey HSD test. Different alphabets represent significant difference. Insect mortality data in percentage, was corrected for mortality in the control using Schneider-Orelli's formula (Püntener, 1981), arcsine transformed and subjected to one-way ANOVA. For nematode survival on leaf, the survival values were arcsine –transformed and subjected to standard least squares by restricted maximum likelihood (REML) followed by Tukey HSD test. Nematode survival (%) (n = 100), insect Mortality (%) of 85%RH (n = 50) and 64%RH (n = 50) were arcsine square-root transformed, subjected to two-way ANOVA and one-way ANOVA respectively. When ANOVA F was significant (p < 0.05), means were compared. Mean was compared using Tukey HSD test. Different alphabets represent significant difference. To identify the wavelength that were strongly affected by humidity, FTIR spectral wavelength data at different humidities and time points were subjected to two-way ANOVA with FDR correction applied to p values.

### Ethics approval

All experimental protocols involving plant materials was conducted in accordance with institutional, national, and international guidelines and legislation under the methods section of the main manuscript file.

## Supplementary Information

Supplementary Information.
