# Supplementary material for: Survival and efficacy of entomopathogenic nematodes on exposed surfaces
Source: Sci Rep. 2022 Mar 17;12:4629. doi: 10.1038/s41598-022-08605-2 (PMC8931053; doi:10.1038/s41598-022-08605-2)
Supplement: Supplementary file 1 — Supplementary Information. [file 41598_2022_8605_MOESM1_ESM.docx]

***SUPPLEMENTARY INFORMATION:***

***FTIR fingerprint spectra***

***Results:***

***
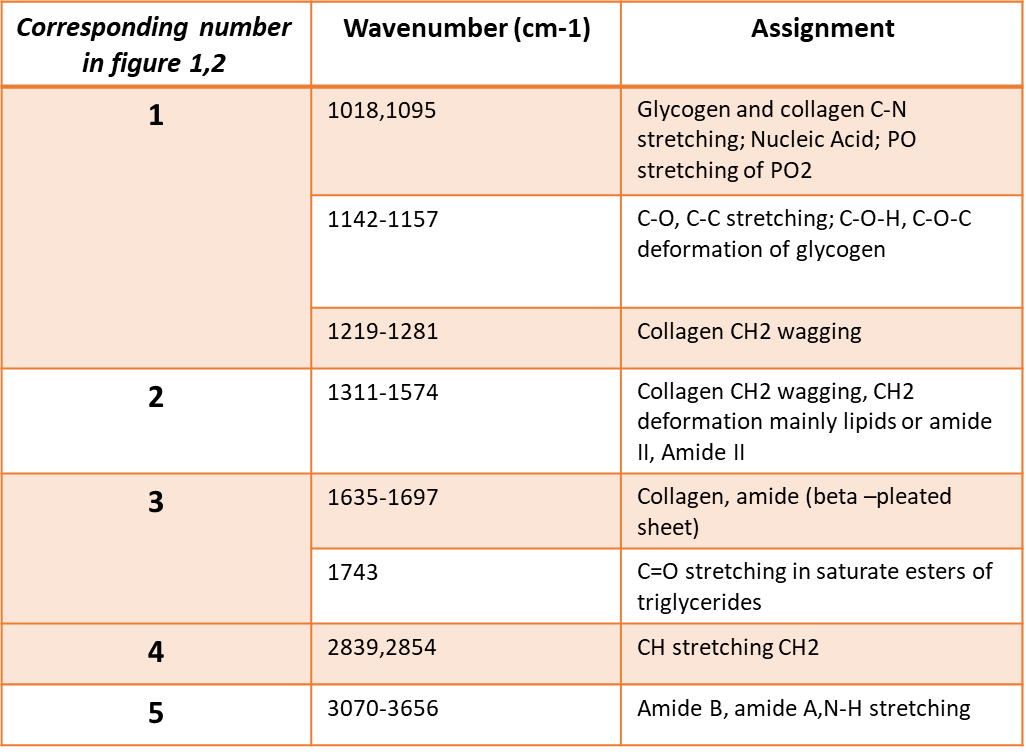
***

***Table S1-*** Assignment of absorption bands in FTIR spectra of *S. carpocapsae*^1^. In addition, two way ANOVA followed by FDR correction on *p* values were applied for *S. carpocapsae* at different humidity and time points highlight these regions that were strongly impacted by water loss.

***In-vitro survival of S. carpocapsae breaking point at lower humidity:***

***Results:***

***
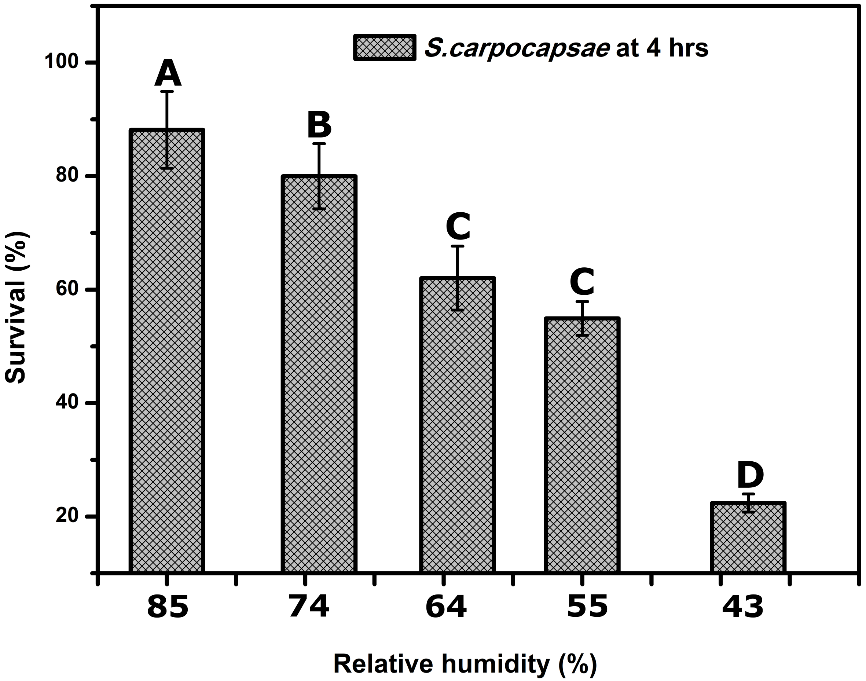
***

**Figure S1:** *In- vitro* survival of *S. carpocapsae* rapidly desiccated at various humidity (85, 74, 64, 55 and 43% RH) for 4 hours. *S. carpocapsae* were evaluated for its breaking point to identify the humidity that caused the maximum reduction in survival.

*S. carpocapsae* IJ were rapidly desiccated at different humidity (85, 74, 64, 55 and 43% RH) for 4 hours. Our results indicate a drastic two-fold reduction in survival from 58% to 20 % between humidity 55-43 %RH respectively. This indicates that, at RD, *S. carpocapsae* IJ survival observes a drastic decline below the humidity of 55% RH.

***FTIR spectra of EPN species RD at 85 % RH***

***Lipid packing changes in EPN at 85 %RH RD (3000-2825 cm^-1^)***

***Results:***

Difference in response of rapidly desiccated *S. carpocapsae* spectral region for symmetric and asymmetric stretching of CH_2_ in lipid chains from *S. feltiae* and *H. bacteriophora* at 85 %RH for 2, 4 hours.

***
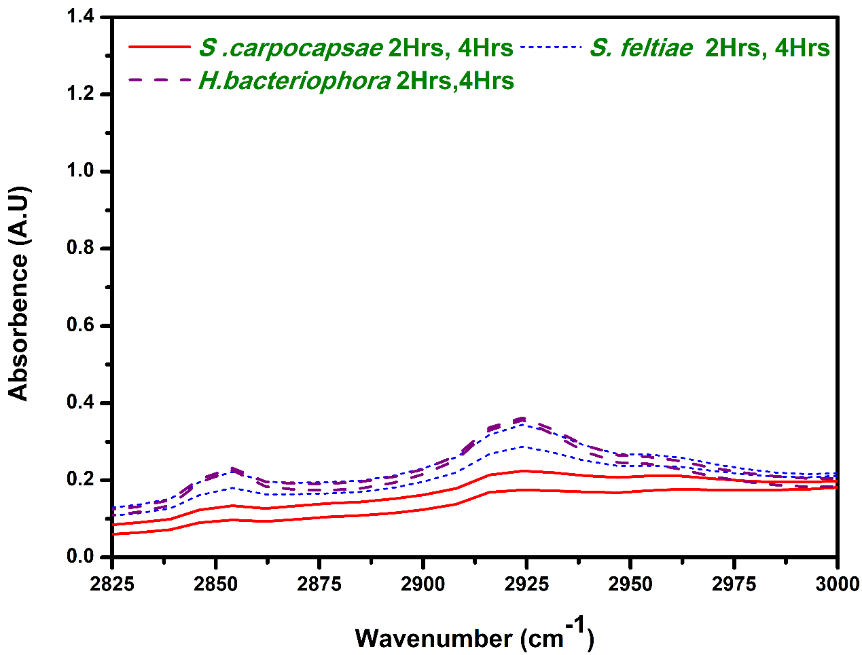
***

***Figure S2-*** FTIR spectra representing the lipid packing changes at region of 2854 and 2924 cm^-1^ as observed in *S. carpocapsae*, *S. feltiae* and *H. bacteriophora* after RD at 85 %RH for 2 and 4 h respectively.

***Principal Component Analysis:***

***Results:***

The Principal component analysis (PCA) of rapidly desiccated EPN species *S. carpocapsae*, *S. feltiae* and *H. bacteriophora* indicate that the response of *S. carpocapsae* is different from *S. feltiae* and *H. bacteriophora* at 4 hours indicated by the black arrows (Figure S3).

***
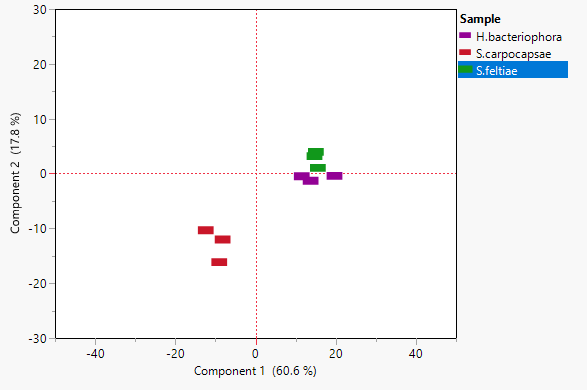
***

***Figure S3-*** Principal Component analysis of EPN species (*S. carpocapsae, S. feltiae and H. bacteriophora)* rapidly desiccated for 4 hours at 85% RH arising from the wavelengths from FTIR spectra.

***
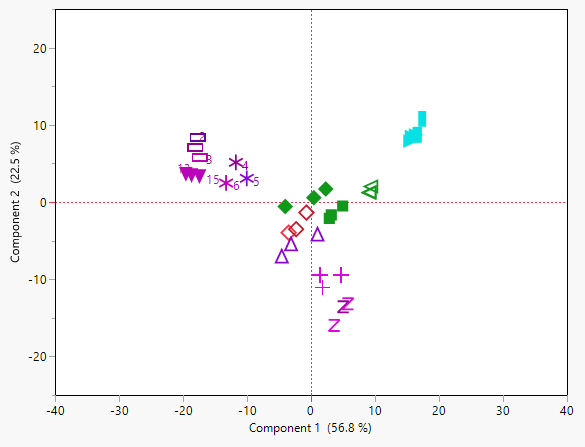
*** ***
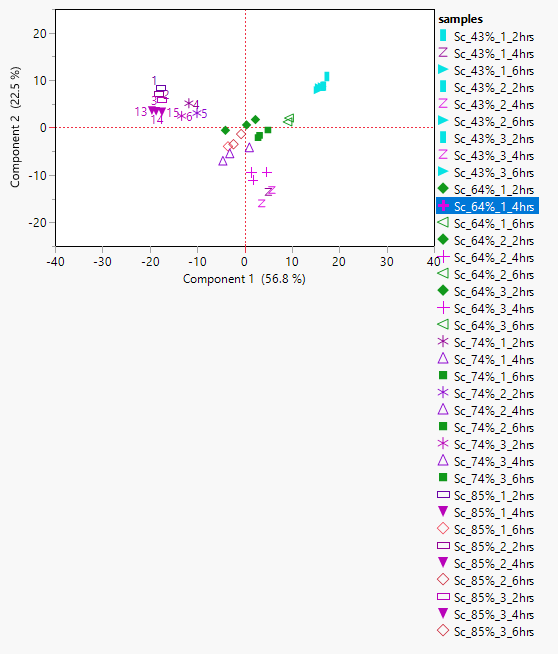
***

***Figure S4-*** Principal Component analysis of *S. carpocapsae* rapidly desiccated for 0, 2, 4, 6 hours at 85, 74, 64 and 43% RH arising from the wavelengths from FTIR spectra.

***Partial least squares-Regression***

***Comparison of actual vs predicted values obtained from Gravimetric estimations and PLS-R predicted values***

***
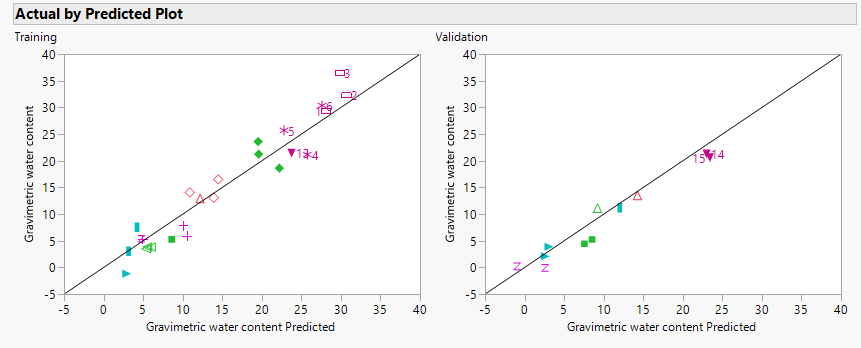
***

***Figure S5-*** PLS-R predictions of water content obtained from FTIR multivariate were compared to gravimetric estimations. Cross validation performed for data obtained from rapidly desiccated *S. carpocapsae* for 0, 2, 4 and 6 hours at 85, 74, 64 and 43% RH.

***References:***

1. San-Blas, E. *et al.* ATR/FTIR characterization of *Steinernema glaseri* and *Heterorhabditis indica*. *Vibrational Spectroscopy* **57**, 220–228 (2011).
